# Supplementary material for: The Evolution and Characterization of the RNA Interference Pathways in Lophotrochozoa
Source: Genome Biol Evol. 2024 May 7;16(5):evae098. doi: 10.1093/gbe/evae098 (PMC11114477; doi:10.1093/gbe/evae098)
Supplement: evae098_Supplementary_Data [file evae098_supplementary_data.zip › Supplementary_materials.zip!/selected_miRNAs/DaRe_selected_miRNas/DaRe_NC_007115.7_5017.pdf]

Provisional ID	:	NC_007115.7_5017
Score total	:	54.8
Score for star read(s)	:	3.9
Score for read counts	:	47
Score for mfe	:	2.8
Score for randfold	:	1.6
Score for cons. seed	:	-0.6
Total read count	:	104
Mature read count	:	81
Loop read count	:	0
Star read count	:	23

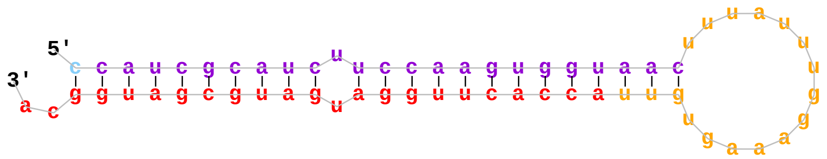

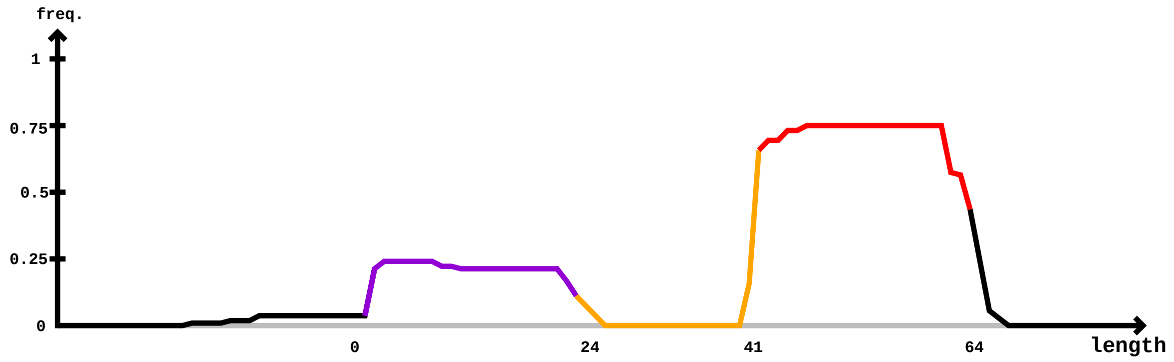


```
                                  Star                                       Mature
5'- cgauaugaauucgccauuguccuguggcugccaucgcaucuuccaagugguaacuuuauuuggaaaguguuaccacuuggaugaugcgauggcagccacaggacaacaucu  -3'   obs
    cgauaugaauucgccauuguccuguggcugccaucgcaucuuccaagugguaacuuuauuuggaaaguguuaccacuuggaugaugcgauggcagccacaggacaacaucu        exp
    (((.......)))...((((((((((((((((((((((((.(((((((((((((..............))))))))))))).)))))))))))))))))))))))).....   reads   mm   sample
    ...............................caucgcaucuuccaaguggu............................................................   1       0    Gu2
    ...............................caucgcaucuuccaaguggua...........................................................   1       0    Gu2
    ......................................................................uaccacuuggaugaugcgaug....................   1       0    Gu2
    ......................................................................uaccacuuggaugaugcgauggA..................   1       1    Gu2
    .......................................................................accacuuggaugaugcgauggc..................   2       0    Gu2
    .......................................................................accacuuggaugaugcgauggcU.................   2       1    Gu2
    .......................................................................accacuuggaugaugcgauggcG.................   1       1    Gu2
    ..........................................................................acuuggaugaugcgaCggca.................   1       1    Gu2

    ............gccUuuguccuguggcugcc...............................................................................   1       1    Gu1
    ...................uccuguggcugcAaucgca.........................................................................   2       1    Gu1
    ...............................caucgcaucuuccaagugguaU..........................................................   1       1    Gu1
    ................................aucgcaucuuccaagugguaac.........................................................   1       0    Gu1
    ......................................................................uaccacuuggaugaugcgaug....................   1       0    Gu1
    ......................................................................uaccacuuggaugaugcgauggU..................   1       1    Gu1
    .......................................................................accacuuggaugaugcgaug....................   1       0    Gu1
    .......................................................................accacuuggaugaugcgauggc..................   2       0    Gu1
    .......................................................................accacuuggaugaugcgauggA..................   1       1    Gu1
    .......................................................................accacuuggaugaugcgauggcC.................   1       1    Gu1
    .......................................................................accacuuggaugaugcgauggca.................   1       0    Gu1

    ...............................caucgcaucuuccaaguggu............................................................   3       0    Ov3
    ...............................caucgcaucuuccaaguggua...........................................................   1       0    Ov3
    ...............................caucgcaucuuccaagugguaC..........................................................   1       1    Ov3
    ...............................caucgcaucuuccaagugguaa..........................................................   1       0    Ov3
    ...............................caucgcaucuuccaagugguaCc.........................................................   1       1    Ov3
    ................................aucgcaucuuccaagugguaac.........................................................   1       0    Ov3
    ......................................................................uaccacuuggaugaugcgaug....................   3       0    Ov3
    .......................................................................accacuuggaugaugcgaug....................   2       0    Ov3
    .......................................................................accacuuggaugaugcgauggca.................   2       0    Ov3
    .......................................................................accacuuggaugaugcgauggcC.................   14      1    Ov3
    .......................................................................Uccacuuggaugaugcgauggcag................   2       1    Ov3
    .......................................................................Uccacuuggaugaugcgauggcagc...............   1       1    Ov3
```

```
                               Star                                    Mature
cgauaugaauucgccauuguccuguggcugccaucgcaucuuccaagugguaacuuuauuuggaaaguguuaccacuuggaugaugcgauggcagccacaggacaacaucu
........................................................................ccacuuggaugaugcgaug....................   2   0   Ov3
............................................................................uuggaugaugcgaCggcagc...............   1   1   Ov3

...............................caucgcaucuuccaaguggua...........................................................   1   0   Ov1
...............................caucgcaucuuccaagugguaCc.........................................................   1   1   Ov1
................................aucgcaucuuccaaguggua...........................................................   1   0   Ov1
................................aucgcaucuuccaagugguaa..........................................................   1   0   Ov1
......................................................................uaccacuuggaugaugcgaug....................   3   0   Ov1
......................................................................uGccacuuggaugaugcgaug....................   1   1   Ov1
......................................................................uaccacuuggaugaugcgauggc..................   1   0   Ov1
.......................................................................Uccacuuggaugaugcgaug....................   1   1   Ov1
.......................................................................accacuuggaugaugcgaug....................   1   0   Ov1
.......................................................................accacuuggaugaugcgauggc..................   2   0   Ov1
.......................................................................accGcuuggaugaugcgauggc..................   1   1   Ov1
.......................................................................accacuuggaugaugcgauggca.................   1   0   Ov1
.......................................................................accacuuggaugaugcgauggcC.................   6   1   Ov1
........................................................................ccacuuggaugaugcgaCggcagcc..............   1   1   Ov1
..........................................................................acuuggaugaugcgaCggcagcc..............   1   1   Ov1

.......................................................................accacuuggaugaugcgauggc..................   1   0   Gu3
.......................................................................accacuuggaugaugcgauggcC.................   1   1   Gu3
........................................................................ccacuuggaugaugcgaCggc..................   1   1   Gu3

................uuguccuguggcugccGucgcauc.......................................................................   1   1   Ov2
...............................caucgcaucuuccaaguggu............................................................   1   0   Ov2
...............................caucgcaucuuccaaguggua...........................................................   2   0   Ov2
...............................caucgcaucuuccaagugguaCc.........................................................   4   1   Ov2
......................................................................uaccacuuggaugaugcgaug....................   3   0   Ov2
......................................................................uaccacuuggaugaugcgaugg...................   1   0   Ov2
......................................................................uaccacuuggaugaugcgauggA..................   1   1   Ov2
.......................................................................accacuuggaugaugcgauggcC.................   8   1   Ov2
..........................................................................acuuggaugaugcgaCggcag................   1   1   Ov2
..........................................................................acuuggaugaugcgaCggcagcc..............   1   1   Ov2
............................................................................uuggaugaugcgaCggcagc...............   1   1   Ov2
```
